# The risk of preterm labor after COVID-19 vaccination before and during pregnancy

**DOI:** 10.3389/fdsfr.2023.1235051

**Published:** 2023-08-25

**Authors:** M. de Feijter, L. C. M. Vissers, L. Davidson, A. C. Kant, P. J. Woestenberg

**Affiliations:** ^1^ Netherlands Pharmacovigilance Centre Lareb, 's-Hertogenbosch, Netherlands; ^2^ Department of Clinical Pharmacology and Toxicology, Leiden University Medical Centre, Leiden, Netherlands

**Keywords:** COVID-19, vaccination safety, teratology, pregnancy, preterm labor, prospective cohort

## Abstract

**Background:** Pregnant women have a higher risk of severe illness and adverse pregnancy outcomes due to a SARS-CoV-2 infection. COVID-19 vaccination can prevent (severe) infection. Observational studies are needed to ascertain safety of COVID-19 vaccination during pregnancy.

**Aim:** Estimate whether COVID-19 vaccination during pregnancy is associated with the risk of preterm labor (PL).

**Methods:** In this prospective cohort study, we included 5,910 pregnant women (mean age: 33.0 ± 3.7 years) who entered the Dutch Pregnancy Drug Register between February 2021 and August 2022. Information on COVID-19 vaccinations, PL, and confounders were self-reported using web-based questionnaires. The hazard ratio (HR) on PL, comparing those who received ≥1 COVID-19 vaccine during any moment of pregnancy to those who did not, was estimated using survival analyses with vaccination as time-varying exposure. Additionally, we estimated the risk of PL after COVID-19 vaccination prior to pregnancy, and after COVID-19 vaccination during trimester 1, 2, or 3 of pregnancy.

**Findings:** A total of 5,227 (88%) participants received ≥1 COVID-19 vaccine between gestational week 2 and 37. We observed no statistically significant association of COVID-19 vaccination during pregnancy (adjusted HR = 0.93, 95%CI = 0.59; 1.45) nor of COVID-19 vaccination prior to pregnancy (adjusted HR = 1.09, 95%CI = 0.70; 1.71) with the risk of PL. Moreover, we observed no association between the risk of PL and COVID-19 vaccination in any trimester of pregnancy.

**Discussion:** We demonstrated that COVID-19 vaccination prior to or during pregnancy is not associated with an increased risk of PL.

**Conclusion:** These results add to the growing evidence supporting safety of COVID-19 vaccination during pregnancy.

## Introduction

When infected with SARS-CoV-2, pregnant women are at higher risk of severe illness and hospital admission as compared to non-pregnant women of reproductive age (Zambrano et al., 2020). Additionally, there is an increased risk of negative pregnancy outcomes or neonatal health problems (Narang et al., 2020). Even asymptomatic infections have been related to adverse outcomes, such as pre-eclampsia or preterm labor (PL) (Watanabe et al., 2022). These negative outcomes can be mitigated by preventing (severe) SARS-CoV-2 infections using COVID-19 vaccinations (Hameed et al., 2022; Watanabe et al., 2022; Zheng et al., 2022). Unfortunately, research to ascertain vaccine safety during pregnancy is limited since pregnant women could not be included in randomized clinical trials due to ethical objections (Watanabe et al., 2022). As a consequence, worldwide vaccination rates in pregnant women are relatively low (Badell et al., 2022).

PL is one of the main outcomes of interest to take in to account when studying vaccine safety during pregnancy (Quinn et al., 2016). Approximately 11% of the pregnancies result in PL (Walani, 2020), which is defined as a livebirth between gestational week 24 and 37 (Quinn et al., 2016). Prematurity is the leading cause of death among children under 5 years of age. Moreover it has been related to both mental and physical disabilities, with more severe complaints for extremely prematurely born children (Quinn et al., 2016; Walani, 2020). Thus far, studies investigating the association of COVID-19 vaccination during pregnancy with PL have been reassuring (Badell et al., 2022). However, most previous studies were based on small samples sizes (Beharier et al., 2021; Bookstein Peretz et al., 2021; Gray et al., 2021), lacked an unvaccinated control group (Bookstein Peretz et al., 2021; Gray et al., 2021; Shimabukuro et al., 2021; Trostle et al., 2021), did not take into account the impact of timing of vaccination in their analyses (Dick et al., 2022; Goldshtein et al., 2022), or had a limited number of exposed participants (Theiler et al., 2021). Therefore, the association between COVID-19 vaccination before or during pregnancy and PL remains largely unclear and more research is needed to ascertain safety of COVID-19 vaccines during pregnancy. Since different biological mechanisms are at play in each trimester of pregnancy, timing of COVID-19 vaccination can possibly influence the association with PL. Studies investigating the risk of PL after COVID-19 vaccination before and during pregnancy are needed, including differences in the risk of PL after vaccination in different trimesters.

Using data from the large Dutch Pregnancy Drug register, we aimed to determine the associations of COVID-19 vaccination before pregnancy and of COVID-19 vaccination during pregnancy with PL. Additionally, we aimed to determine whether the association of COVID-19 vaccination with PL differed between the trimesters of pregnancy.

## Methods

### Study design

This study used data from the Dutch Pregnancy Drug Register. This ongoing cohort of pregnant women living in the Netherlands was set up in 2014 with the main aim of pharmacovigilance by determining use of medication during pregnancy and examining the safety of medication exposure in relation to pregnancy-related outcomes. Information on general health, lifestyle, medication exposure, course of the pregnancy, child birth, and child health is collected using web-based questionnaires. Pregnant women could start participation at any time during pregnancy and received up to three questionnaires during pregnancy (enrollment, 17 and 34 weeks of gestation) and three questionnaires after birth (2, 6, and 12 months postpartum). Vorstenbosch and others previously described details of the study design (Vorstenbosch et al., 2019). All procedures performed were in accordance with ethical standards of the institutional or national research committee and with the 1964 Helsinki declaration and its later amendments or comparable ethical standards. The Regional Committee on Research Involving Human Subjects, Arnhem-Nijmegen judged in 2013 that the Dutch Pregnancy Drug Register does not require specific ethical approval since it collects data by means of questionnaires only (protocol number 2013/259). In June 2022 the Medical Ethical Committee Brabant judged this was still applicable (protocol number NW 2022-41). All participants gave informed consent digitally.

### Assessment of COVID-19 vaccination (exposure)

In each questionnaire participants were asked whether they had received one or multiple COVID-19 vaccines since the start of pregnancy (baseline questionnaire) or since the last questionnaire (follow-up questionnaires). In case participants reported a COVID-19 vaccination, we additionally asked when this vaccination was given (reported as a date or gestational week) and the brand of the vaccine. In the first questionnaire participants were additionally asked whether or not they received a COVID-19 vaccination in the year before conception. Based on this information we established different exposure variables, which are independent of each other.

#### COVID-19 vaccination during pregnancy

A time-varying variable, where exposure is defined as receiving ≥1 COVID-19 vaccination between gestational week 2 and 37. To overcome immortal time bias (Suissa, 2008), where unexposed time falsely contributes to exposed time, we used a time-varying approach. The timing of the first COVID-19 vaccination during pregnancy was used as moment of exposure, where the time before this vaccine was interpreted as non-exposed time, irrespective of potential COVID-19 vaccination(s) prior to pregnancy. Time from the moment of this first COVID-19 vaccine during pregnancy onwards contributes as exposed.

#### COVID-19 vaccination prior to pregnancy

A binary variable (“*yes*” or “*no*”), where exposure is defined as receiving ≥1 COVID-19 vaccination in the year prior to pregnancy. Vaccinations that were received before gestational week 2 were considered as vaccines prior to pregnancy.

#### COVID-19 vaccination during trimester 1

A binary variable (“*yes*” or “*no*”), where exposure is defined as receiving ≥1 COVID-19 vaccination between gestational week 2 and 12.

#### COVID-19 vaccination during trimester 2

A binary variable (“*yes*” or “*no*”), where exposure is defined as receiving ≥1 COVID-19 vaccination between gestational week 12 and 26.

#### COVID-19 vaccination during trimester 3

A time-varying variable where exposure is defined as receiving ≥1 COVID-19 vaccination between gestational week 26 and 37. The timing of the first COVID-19 vaccination during trimester 3 of pregnancy was used as moment of exposure. The time before this vaccine was interpreted as non-exposed time, irrespective of potential COVID-19 vaccination(s) prior to pregnancy, or during trimester 1 or 2 of pregnancy. Time from the moment of this first COVID-19 vaccine during trimester 3 pregnancy onwards contributes as exposed.

### Assessment of preterm labor (outcome)

PL was defined as a livebirth between gestational week 24 and 37 (Quenby et al., 2021). Gestational age at delivery was based on the estimated date of delivery (based on ultrasound) or the first day of the last menstrual period.

### Other variables

As potential confounders we selected *a priori*: maternal and paternal age at the estimated date of delivery, highest level of education of both biological parents based on the UNESCO International Standard Classification of Education (United Nations Educational, 1976) (“*not high educated*,” “*one parent high educated*,” or “*both high educated*”) as proxy for social economic status, level of urbanicity [5 levels using the zip code (2021)], maternal alcohol consumption and smoking behavior since 3 months prior to or during pregnancy (“*No*,” “*Discontinued at conception*,” “*Discontinued at positive pregnancy test*,” or “*Continued during pregnancy*,”) maternal illicit drug use since 3 months prior to conception or during pregnancy (“*No*” or “*Yes*”), pre-pregnancy Body Mass Index (kg/m^2^), history of preterm labor (“*No*,” “*Yes*,” or “*No previous delivery*”), vaccination priority (“*No*” or “*Yes*”), and pregnancy start month as a proxy for impact of pandemic-related restrictions, risk on SARS-CoV-2 infections, and vaccination availability. Vaccination priority was based on recommendations from the Dutch National Institute for Public Health and the Environment, stating that people with chronic respiratory conditions, chronic heart disease, kidney disease, diabetes, morbid obesity, and immune deficiency were strongly recommended to get vaccinated, irrespective of a potential pregnancy. Hypertension, pre-eclampsia, gestational diabetes, polyhydramnios, and oligohydramnios are pregnancy complications that can result in medically induced preterm labor. In case participants reported any of these complications, the variable pregnancy complications was set to “*Yes*”. Additionally, we estimated whether participants suffered from a SARS-CoV-2 infection between gestational week 2 and 37 (“*No*” or “*Yes*”), where timing of infection was based on the positive (self-)test result.

### Participants

Between February 2021 and August 2022, 10,698 women filled out the first questionnaire. For this study we selected women who entered the Dutch Pregnancy Drug Register before gestational week 37, and had been pregnant for ≥24 weeks (N = 6,782). We excluded participants who had no follow-up data (N = 629), reported a twin pregnancy (N = 76), reported a stillbirth (N = 8), had an unclear vaccination status (N = 15), or unknown vaccination timing (N = 144), leaving 5,910 eligible study participants.

A participant contributes observation time to our analyses from gestational week 24 onwards. If entry into our cohort was after gestational week 24, we had to take into account left truncation (Howards et al., 2007). To overcome left truncation bias these participants started contributing observation time to our analyses from the moment of inclusion in our cohort. Furthermore we estimated whether left truncation was non-informative by estimating the association between any of the confounders and enrollment time using regression analyses (Xu et al., 2012). End of contribution was set to gestational week 37 or moment of delivery (when prior to gestational week 37). Participants who did not have complete follow-up were censored at the gestational age of their last questionnaire.

### Statistical analyses

We presented the descriptive statistics for categorical variables as number with percentage and for continuous data as mean with standard deviation (SD). For covariables, missing values were handled by using multiple imputation with 5 imputation sets and 20 iterations, using the MICE R package (Buuren and Groothuis-Oudshoorn, 2010).

The association between COVID-19 vaccination status and PL was prospectively assessed through Kaplan-Meier curves and via univariable and multivariable Cox proportional-hazards regression models. Risks were expressed as Hazard Ratios (HR) with 95% confidence intervals (95%CI), where we checked the proportional-hazard assumption using log–log plots and testing Schoenfeld residuals (Zhang et al., 2018).

#### COVID-19 vaccination during and prior to pregnancy

We studied COVID-19 vaccination during pregnancy as a time-varying variable to overcome immortal time bias. Exposure prior to pregnancy was studied as a binary variable. The crude HR was studied for COVID-19 vaccination during pregnancy and for COVID-19 vaccination prior to pregnancy in separate models (model 0). Furthermore, we estimated the adjusted HR in a model including both COVID-19 vaccination during pregnancy and COVID-19 vaccination prior to pregnancy (model 1). Model 2 additionally adjusted for maternal age, paternal age, parental level of education, and urbanicity and model 3 additionally adjusted for maternal age, paternal age, parental level of education, urbanicity, smoking behavior, intake of alcohol, use of illicit drugs, pre-pregnancy body mass index, pregnancy complications, history of PL, vaccination priority mother, and pregnancy start month.

To estimate the robustness of our results, we performed multiple sensitivity analyses for COVID-19 vaccination during pregnancy and COVID-19 vaccination prior to pregnancy. First, to limit potential left truncation bias due to delayed enrollment in the cohort, we repeated our analyses on excluding those who started participating after gestational week 24. Second, to overcome the potential impact of a SARS-CoV-2 infection on PL we repeated our analyses after excluding all participants who had a SARS-CoV-2 infection whilst pregnant. Third, to estimate the potential impact of COVID-19 vaccination prior to pregnancy on the HR of vaccination during pregnancy, we repeated our analyses after excluding those with a COVID-19 vaccination prior to pregnancy. Fourth, women with medically induced labor before gestational week 37 were excluded from the sample. Last, to overcome potential differences between vaccine brands, we repeated our analyses after excluding those who received a vector vaccine, or with an unknown brand.

#### COVID-19 vaccination per trimester of pregnancy

Because During different trimesters of pregnancy different biological mechanisms are at play, meaning that the mechanism of a potential effect of COVID-19 vaccination on PL might also be different for each trimester. For example, a COVID-19 vaccination during trimester 3 might trigger PL due to reactogenicity of vaccination, while vaccination during trimester 1 would be a long-term effect. Because these potential differences in risk are omitted when studying COVID-19 vaccination exposure at any time during pregnancy, we estimated the association between COVID-19 vaccination and the risk of PL during different trimesters.

In these analyses we studied exposure during trimester 1 and 2 as binary variables. This was done because for exposure during trimester 1 and 2, exposure was by definition before the outcome (PL) and because a potential effect of COVID-19 vaccination during trimester 1 and 2 on PL risk is mostly a long-term effect. Exposure during trimester 3 was studied as a time-varying variable, to overcome immortal-time-bias. We estimated the crude HR for COVID-19 vaccination during trimester 1, during trimester 2, and during trimester 3 separately. Afterwards, again, the adjusted HR was estimated in models 1, 2 and 3. Where in each model we included COVID-19 vaccination during trimester 1, during trimester 2, and during trimester 3 together and additionally adjusted for COVID-19 vaccination prior to pregnancy.

All analyses were performed in R version R 4.1.3 (R Foundation for Statistical Computing, Vienna, Austria, www.R-project.org) with a statistical significance level of *p* < 0.05.

## Results

In total 5,910 women (mean age: 33.0 ± 3.7) contributed to our analyses, of whom 5,227 (88%) received at least one COVID-19 vaccination between gestational week 2 and 37 (see Table 1). Most women reported they were vaccinated with a mRNA vaccine from Pfizer-BioNTech (BNT162b2, 85%) or Moderna (mRNA-1273, 12%). A small percentage of women reported they were vaccinated with a vector vaccine from Oxford-AstraZeneca (ChAdOx1 nCoV-19, 1%) or Janssen (Ad26.COV2.S, <1%). For approximately 1%, the type of vaccine was unknown. Of the 5,227 women who received a COVID-19 vaccination during pregnancy, 1,526 (29%) also received ≥1 COVID-19 vaccination prior to pregnancy. Of the 683 women who did not receive a COVID-19 vaccination during pregnancy, 251 (37%) did receive ≥1 COVID-19 vaccinations prior to pregnancy. Within our cohort, we observed no association between any of the confounders and enrollment time (data not shown), indicating that left truncation is largely non-informative (Xu et al., 2012).

**TABLE 1:** Baseline characteristics of the study population.

	Total	Not vaccinated^a^	Vaccinated^a^
N	5,910	683	5,227
Preterm labor current pregnancy	179 (3%)	22 (3%)	157 (3%)
Age mother (years)	33.0 ± 3.7	32.0 ± 3.9	33.1 ± 3.7
Age father (years)	35.2 ± 4.8	34.3 ± 5.0	35.3 ± 4.8
Education parents			
Both high educated	3,981 (67%)	340 (50%)	3,641 (70%)
One high educated	1,260 (21%)	193 (28%)	1,067 (20%)
Not high educated	470 (8%)	129 (19%)	341 (7%)
Ethnicity parents			
Both Dutch	4,935 (84%)	578 (85%)	4,357 (83%)
One Dutch	565 (10%)	56 (8%)	509 (10%)
Not Dutch	260 (4%)	35 (5%)	225 (4%)
Urbanicity			
Very high	1,540 (26%)	133 (20%)	1,407 (27%)
High	1,507 (26%)	156 (23%)	1,351 (26%)
Moderately high	1,056 (18%)	138 (20%)	918 (18%)
Low	1,016 (17%)	152 (22%)	864 (17%)
Very low	767 (13%)	101 (15%)	666 (13%)
Smoking behaviour mother^b^			
No smoking	5,410 (92%)	588 (86%)	4,822 (92%)
Stopped at conception	269 (5%)	51 (8%)	218 (4%)
Stopped at positive pregnancy test	108 (2%)	14 (2%)	94 (2%)
Continued smoking during pregnancy	86 (1%)	24 (4%)	62 (1%)
Alcohol intake mother^b^			
No alcohol	1,424 (24%)	186 (27%)	1,238 (24%)
Stopped at conception	3,416 (58%)	382 (56%)	3,034 (58%)
Stopped at positive pregnancy test	825 (14%)	87 (13%)	738 (14%)
Continued alcohol during pregnancy	210 (4%)	24 (4%)	186 (4%)
Illicit drug use mother^b^	164 (3%)	20 (3%)	144 (3%)
Body mass index^c^ (kg/m^2^)	24.2 ± 4. 5	24.5 ± 5.0	24.2 ± 4.5
Pregnancy complication	995 (17%)	100 (15%)	895 (17%)
History of preterm labor			
No previous delivery	4,346 (74%)	495 (73%)	3,851 (74%)
No	1,450 (24%)	166 (24%)	1,284 (25%)
Yes	99 (2%)	17 (3%)	82 (2%)
SARS-CoV-2 infection during pregnancy	1,222 (21%)	260 (38%)	962 (18%)
Priority group for vaccination	954 (16%)	95 (14%)	859 (17%)
Vaccination prior to pregnancy	1,774 (30%)	250 (37%)	1,524 (29%)

Abbreviations: SD, standard deviation. For categorical variables the absolute number (%) is indicated, for numeric variables the mean ± SD., Missing values: age father (*n* = 206, 4%), education (*n* = 199, 3%), ethnicity (*n* = 150, 3%), urbanicity (*n* = 24, 0.4%), smoking (*n* = 37, 0.6%), alcohol (*n* = 34, 0.6%), drugs (*n* = 21, 0.4%), body mass index (*n* = 388, 7%), and SARS-CoV-2, infection during pregnancy (*n* = 474, 8%). For other variables there are no missing values.

^a^ Based on COVID-19, vaccination between gestational week 2 and 37.

^b^ Assessed since 3 months prior to conception.

^c^ Based on body weight prior to pregnancy.

### COVID-19 vaccination during and prior to pregnancy

**FIGURE 1:**
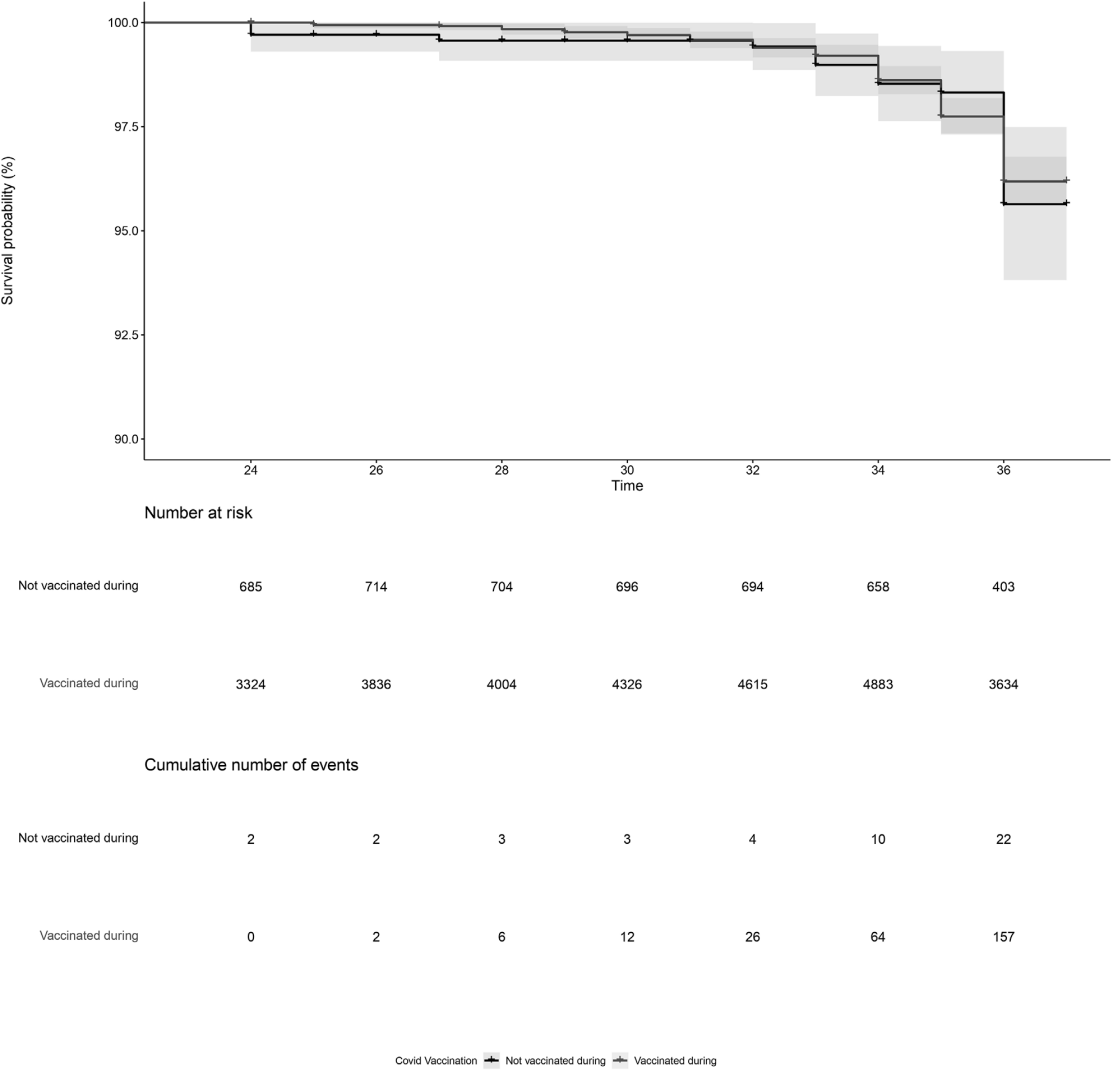
Kaplan-Meier plot of time (gestational week) by COVID-19 vaccination status, for vaccination during pregnancy. Not vaccinated during pregnancy (black) indicates the participant did not receive a COVID-19 vaccine between gestational week 2 and 37. Vaccinated during pregnancy (grey) indicates the participant received a COVID-19 vaccination between gestational week 2 and 37. Participants start contributing to the plot from the gestational week at the moment of inclusion in our cohort. Vaccination status during pregnancy was time-dependent, meaning that a woman could contribute observation time to the unvaccinated and the vaccinated observation time if she received a COVID-19 vaccination between gestational week 2 and 37.

**FIGURE 2:**
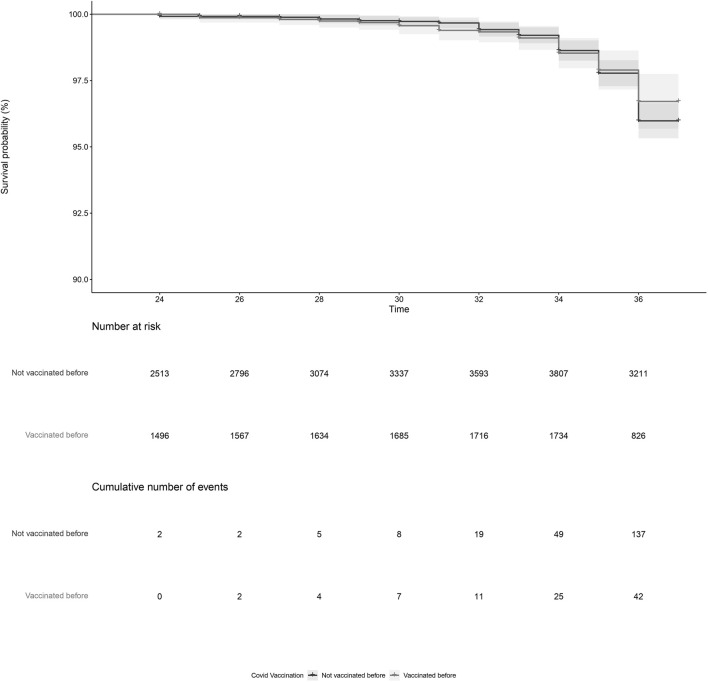
Kaplan-Meier plot of time (gestational week) by COVID-19 vaccination status, for vaccination prior to pregnancy. Not vaccinated before pregnancy (black) indicates the participant did not receive a COVID-19 vaccine prior to pregnancy. Vaccinated before pregnancy (grey) indicates the participant received a COVID-19 vaccination prior to pregnancy. Participants start contributing to the plot from the gestational week at the moment of inclusion in our cohort.

PL was reported by 179 women, of which 157 were vaccinated and 22 were not vaccinated during pregnancy. After adjustment, COVID-19 vaccination during pregnancy (adjusted HR = 0.93, 95%CI = 0.59; 1.45, Figure 1; Table 2, model 3) and COVID-19 vaccination prior to pregnancy (adjusted HR = 1.09, 95%CI = 0.70; 1.71, Figure 2 and Table 2, model 3) were not statistically significant associated with PL. Our sensitivity analyses did not affect our findings substantially (Supplementary Table S1). We did not observe any structural patterns in the timing between vaccination and PL (Supplementary Table S2).

**TABLE 2:** Hazard ratio of preterm labor according to COVID-19 vaccination status.

	Model 0 (crude)		Model 1		Model 2		Model 3	
	HR (95% CI)	*p*-value	HR (95% CI)	*p*-value	HR (95% CI)	*p*-value	HR (95% CI)	*p*-value
Vaccination during pregnancy	0.91 (0.58; 1.42)	0.68	0.90 (0.58; 1.41)	0.65	0.89 (0.57; 1.38)	0.60	0.94 (0.60; 1.47)	0.79
Vaccination prior to pregnancy	0.86 (0.61; 1.21)	0.40	0.87 (0.61; 1.21)	0.38	0.87 (0.61; 1.22)	0.42	1.10 (0.70; 1.72)	0.69

Abbreviations: HR, hazard ratio; CI, Confidence Interval. Effect estimates were crude (model 0), a model including COVID-19, vaccination during pregnancy and COVID-19, vaccination prior to pregnancy (model 1); a model including COVID-19, vaccination during pregnancy COVID-19, vaccination prior to pregnancy, age biological mother, age biological father, educational level of biological parents, and level of urbanicity (model 2); a model including COVID-19, vaccination during pregnancy COVID-19, vaccination prior to pregnancy, age biological mother, age biological father, educational level of biological parents, level of urbanicity, smoking behavior, alcohol intake, illicit drug use, pre-pregnancy body mass index, history of preterm birth, pregnancy complication, mother belonging to a high priority group for vaccination, and pregnancy start month (model 3).

### COVID-19 vaccination per trimester of pregnancy

Within our study population, 1,431 (24%) women were vaccinated during trimester 1, 3,059 (52%) during trimester 2, and 2,289 (39%) during trimester 3 of pregnancy (Supplementary Table S3). After adjustment for potential confounders, we observed no association of COVID-19 vaccination in trimester 1 (adjusted HR = 0.72, 95%CI = 0.47; 1.09), trimester 2 (adjusted HR = 1.09, 95%CI = 0.78; 1.53), or trimester 3 (adjusted HR = 0.84, 95%CI = 0.58; 1.21) with PL (Table 3).

**TABLE 3:** Hazard ratio of preterm labor according to COVID-19 vaccination status by trimester.

	Model 0 (crude)		Model 1		Model 2		Model 3	
	HR (95% CI)	*p*-value	HR (95% CI)	*p*-value	HR (95% CI)	*p*-value	HR (95% CI)	*p*-value
Vaccination during trimester 1	0.63 (0.43; 0.93)	0.021	0.65 (0.43; 0.97)	0.037	0.67 (0.44; 1.00)	0.05	0.72 (0.47; 1.09)	0.12
Vaccination during trimester 2	1.20 (0.89; 1.62)	0.24	1.06 (0.76; 1.49)	0.72	1.06 (0.76; 1.49)	0.71	1.09 (0.78; 1.53)	0.61
Vaccination during trimester 3	0.95 (0.69; 1.31)	0.76	0.92 (0.65; 1.31)	0.66	0.90 (0.63; 1.29)	0.57	0.84 (0.58; 1.21)	0.34

Abbreviations: HR, hazard ratio; CI, Confidence Interval. Effect estimates were crude (model 0), a model including COVID-19, vaccination in trimester 1; COVID-19, vaccination in trimester 2; COVID-19, vaccination in trimester 3, and COVID-19, vaccination prior to pregnancy (model 1); a model including COVID-19, vaccination in trimester 1; COVID-19, vaccination in trimester 2; COVID-19, vaccination in trimester 3, and COVID-19, vaccination prior to pregnancy, age biological mother, age biological father, educational level of biological parents, and level of urbanicity (model 2); a model including COVID-19, vaccination in trimester 1; COVID-19, vaccination in trimester 2; COVID-19, vaccination in trimester 3, and COVID-19, vaccination prior to pregnancy, age biological mother, age biological father, educational level of biological parents, level of urbanicity, smoking behavior, alcohol intake, illicit drug use, pre-pregnancy body mass index, history of preterm birth, pregnancy complication, mother belonging to a high priority group for vaccination, and pregnancy start month (model 3). Of the 1,431 women that were vaccinated during trimester 1, 30 reported a preterm labor. For women who were not vaccinated during trimester 1 (*n* = 4,453), 149 reported preterm labor. Of the 3,059 women that were vaccinated during trimester 2, 106 reported a preterm labor. For women who were not vaccinated during trimester 2 (*n* = 2,825), 73 reported preterm labor. Of the 2,289 women that were vaccinated during trimester 3, 60 reported a preterm labor. For women who were not vaccinated during trimester 3 (*n* = 3,595), 119 reported preterm labor.

## Discussion

### Principal findings

This study suggests no association of COVID-19 vaccination during or prior to pregnancy with PL. These conclusions remained similar after multiple sensitivity analyses. Additionally, after studying COVID-19 vaccination in different trimesters of pregnancy we observed no association with PL for COVID-19 vaccination in any of the trimesters.

### Results in the context of what is known

The absence of an association between COVID-19 vaccination and PL in this study is in line with previous literature studying the association of the COVID-19 vaccine (Badell et al., 2022) or other vaccines (Arora and Lakshmi, 2021) with PL. These findings support that COVID-19 vaccination can be safely administered to pregnant women in order to prevent (severe) SARS-CoV-2 infections (Hameed et al., 2022; Watanabe et al., 2022; Zheng et al., 2022). There have only been a few studies reporting an increased (Dick et al., 2022) or decreased (Carbone et al., 2022; Hui et al., 2022; Piekos et al., 2022) risk of PL after COVID-19 vaccination. However, all studies reporting an altered risk on PL after COVID-19 vaccination did not handle time window bias correctly. When, for example, timing of inclusion in the study population, timing of vaccination, or timing of the event are not considered properly this can have serious impact on the effect estimates and therefore lead to false conclusions (Di Martino et al., 2015). Within our study we were able to compare exposed and unexposed individuals from the same target population, correct for possible confounders, and take into account the aspect of time. Reactions reported after COVID-19 vaccinations, both mRNA and vector, were mostly mild and transient (Chapin-Bardales et al., 2021; Menni et al., 2021). Combined with data on other maternal vaccines (Villar et al., 2012; Munoz and Jamieson, 2019), it is therefore unlikely COVID-19 vaccination will introduce risk factors for PL by, for example, affecting the fetus or functioning of the placenta. Together this supports the absence of an association between COVID-19 vaccination before or during pregnancy and PL is plausible.

Presumably, timing of exposure to COVID-19 vaccination during pregnancy could be of influence in a potential association with the risk of PL, because other developmental and biological mechanisms are at play during different trimesters of pregnancy. Thus far most studies were largely based on third-trimester exposure as only a limited number of participants received a COVID-19 vaccine during trimester 1 or 2. Therefore only few studies were able to take into account potential differences between trimesters, with mixed results. Only one study, by Dick et al. (2022), reported an increased risk of PL after COVID-19 vaccination in trimester 2. However, they did not consider any confounding factors. Within our cohort, COVID-19 vaccination was well presented for each trimester, which allowed us to investigate potential effect of timing of vaccination during pregnancy. We were able to estimate the risk of PL after COVID-19 vaccination in different trimesters and take into account confounders and the impact of timing.

### Clinical implications

We observed that exposure to COVID-19 vaccination was not associated with an altered risk on PL in any of the trimesters of pregnancy. These findings implicate that COVID-19 vaccination during pregnancy is not associated with a long-term or short-term risk of PL. The absence of an association of COVID-19 vaccination in early pregnancy with PL suggests there is not likely a teratogenic effects or impact on functioning of the placenta, which could eventually lead to PL (Quinn et al., 2016). Our crude results even suggest COVID-19 vaccination during trimester 1 reduces the risk on PL. It is likely that this association lost significance after multivariate correction due to a lack of power. An explanation for the protective effect of a first trimester COVID-19 vaccination on PL could be the reduced risk on (severe) SARS-CoV-2 infections due to COVID-19 vaccination. SARS-CoV-2 infections have been linked to an increased risk on preterm labor, especially in women with a symptomatic infection or comorbidities (Wei et al., 2021). However, because of the limited number of women that reported a preterm labor and vaccination during trimester 1 these findings should be interpreted with caution. Future studies focusing on exposure to COVID-19 vaccination during trimester 1 are needed to gain insight in the potential associations between COVID-19 vaccination, birth defects, and preterm labor. Additionally, the absence of an association of COVID-19 vaccination in trimester 3 with PL, combined with the absence of a structural pattern in time between moment of vaccination and the time of delivery implicates that COVID-19 vaccination does not increase the risk of PL shortly after exposure.

### Research implications

We observed no association of COVID-19 vaccination prior to or during pregnancy with the risk of PL. However, we were not able to take into account the total number of COVID-19 vaccinations a women received or stratify our analyses for brand of vaccination. Further studies are required to estimate potential differences between different brands of COVID-19 vaccines.

### Strengths and limitations

Several limitations need to be considered when interpreting our results. First, we had incomplete information about COVID-19 vaccinations prior to pregnancy, therefore it was not possible to take into account differences between a first, second or booster dose. Second, because participants could enter our study at any moment during pregnancy we had to deal with left truncation. Nevertheless, after excluding those who entered our study after gestational week 24, our results did not substantially change. Moreover, the data suggested that left truncation was largely non-informative. Third, we had a relatively small control sample of participants who did not receive a COVID-19 vaccination during pregnancy, of whom 37% were vaccinated prior to pregnancy. This group might therefore not be representative for all unexposed pregnant women. Last, even though we had a substantial number of potential confounders that could be included in our models, as for all observational studies, residual confounding could potentially have affected our findings. However, having longitudinal data in a large sample of pregnant women with a substantial level of exposed and unexposed participants is unique in the field and allowed us to prospectively assess associations between COVID-19 vaccination and PL*.*

## Conclusion

Altogether, in our study we did not observe an association between COVID-19 vaccination before or during any trimester of pregnancy and the risk of PL. These findings underwrite safety of COVID-19 vaccination before or during pregnancy.

## Data Availability

The raw data supporting the conclusion of this article will be made available by the authors, without undue reservation.
