# Supplementary material for: The risk of preterm labor after COVID-19 vaccination before and during pregnancy
Source: Front Drug Saf Regul. 2023 Aug 25;3:1235051. doi: 10.3389/fdsfr.2023.1235051 (PMC12443077; doi:10.3389/fdsfr.2023.1235051)
Supplement: Supplementary file 1 [file Table1.DOCX]

**Table S1**. Hazard Ratio of preterm labor according to COVID-19 vaccination status in the sensitivity analyses.

|  | **Women starting participation before 24 weeks gestational age**  **(N = 4,186)** | | **Women without SARS-CoV-2 infection during pregnancy**  **(N = 4,213)** | | **Women not vaccinated against COVID-19 prior to pregnancy**  **(N = 4,114)** | | **Women without medically induced labor before gestational week 37**  **(N = 5,872)** | | **Women vaccinated with an mRNA vaccine, Pfizer or Moderna**  **(N = 5,243)** | |
| --- | --- | --- | --- | --- | --- | --- | --- | --- | --- | --- |
|  | HR  (95% CI) | p-value | HR  (95% CI) | p-value | HR  (95% CI) | p-value | HR  (95% CI) | p-  value | HR  (95% CI) | p-  value |
| **Vaccination during pregnancy** | 0.96 (0.56;1.63) | 0.89 | 0.85 (0.50;1.44) | 0.54 | 0.95 (0.56;1.61) | 0.85 | 0.89 (0.57;1.41) | 0.63 | 0.87 (0.55;1.38) | 0.56 |
| **Vaccination prior to pregnancy** | 1.06 (0.64;1.77) | 0.82 | 1.09 (0.64;1.86) | 0.74 | - | - | 1.06 (0.67;1.68) | 0.80 | 1.10  (0.70;1.27) | 0.69 |

Abbreviations: HR, Hazard Ratio; CI, Confidence Interval. Effect estimates were based on a model including COVID-19 vaccination during pregnancy, COVID-19 vaccination prior to pregnancy, age biological mother, age biological father, educational level of biological parents, level of urbanicity, smoking behavior, alcohol intake, illicit drug use, pre-pregnancy body mass index, history of preterm labor, pregnancy complication, mother belonging to a high priority group for vaccination, and pregnancy start month (model 3).

**Table S2**. Overview of time between COVID-19 vaccination and preterm labor, for those who received ≥1 COVID-19 vaccination during pregnancy and experienced preterm labor (n=157).

| **Last COVID-19 vaccination during pregnancy in** **gestational week** | **Vaccinated prior to pregnancy** | **Number of COVID-19 vaccines during pregnancy** | **Preterm labor in gestational week** | **Time between COVID-19 vaccine and labor (weeks)** |
| --- | --- | --- | --- | --- |
| 36 | No | 2 | 36 | 0 |
| 26 | No | 2 | 27 | 1 |
| 34 | No | 2 | 35 | 1 |
| 33 | No | 1 | 34 | 1 |
| 33 | No | 1 | 34 | 1 |
| 34 | No | 1 | 35 | 1 |
| 35 | No | 3 | 36 | 1 |
| 35 | No | 3 | 36 | 1 |
| 34 | No | 2 | 35 | 1 |
| 28 | No | 2 | 29 | 1 |
| 34 | No | 2 | 36 | 2 |
| 33 | No | 2 | 35 | 2 |
| 33 | No | 2 | 35 | 2 |
| 33 | No | 2 | 35 | 2 |
| 30 | No | 2 | 32 | 2 |
| 32 | No | 1 | 34 | 2 |
| 32 | No | 2 | 34 | 2 |
| 33 | No | 2 | 36 | 3 |
| 33 | No | 1 | 36 | 3 |
| 33 | No | 2 | 36 | 3 |
| 31 | No | 1 | 34 | 3 |
| 33 | No | 1 | 36 | 3 |
| 32 | No | 2 | 35 | 3 |
| 29 | No | 2 | 32 | 3 |
| 28 | Yes | 1 | 31 | 3 |
| 32 | No | 2 | 35 | 3 |
| 32 | No | 1 | 35 | 3 |
| 33 | No | 1 | 36 | 3 |
| 32 | No | 2 | 35 | 3 |
| 32 | No | 2 | 36 | 4 |
| 32 | No | 1 | 36 | 4 |
| 28 | No | 1 | 32 | 4 |
| 31 | No | 2 | 35 | 4 |
| 29 | No | 2 | 33 | 4 |
| 30 | Yes | 3 | 34 | 4 |
| 26 | No | 2 | 31 | 5 |
| 29 | No | 3 | 34 | 5 |
| 29 | No | 1 | 34 | 5 |
| 31 | No | 2 | 36 | 5 |
| 27 | No | 2 | 32 | 5 |
| 31 | No | 2 | 36 | 5 |
| 31 | No | 2 | 36 | 5 |
| 31 | No | 2 | 36 | 5 |
| 24 | No | 1 | 30 | 6 |
| 22 | No | 1 | 28 | 6 |
| 30 | No | 1 | 36 | 6 |
| 28 | No | 1 | 34 | 6 |
| 30 | No | 1 | 36 | 6 |
| 29 | No | 2 | 35 | 6 |
| 30 | No | 3 | 36 | 6 |
| 29 | No | 2 | 35 | 6 |
| 30 | No | 2 | 36 | 6 |
| 29 | No | 2 | 35 | 6 |
| **Last COVID-19 vaccination during pregnancy in** **gestational week** | **Vaccinated prior to pregnancy** | **Number of COVID-19 vaccines during pregnancy** | **Preterm labor in gestational week** | **Time between COVID-19 vaccine and PL (weeks)** |
| 30 | No | 2 | 36 | 6 |
| 30 | No | 2 | 36 | 6 |
| 29 | Yes | 3 | 35 | 6 |
| 24 | No | 2 | 31 | 7 |
| 21 | No | 1 | 28 | 7 |
| 18 | Yes | 1 | 25 | 7 |
| 23 | Yes | 1 | 30 | 7 |
| 29 | Yes | 1 | 36 | 7 |
| 29 | No | 2 | 36 | 7 |
| 29 | No | 2 | 36 | 7 |
| 29 | No | 2 | 36 | 7 |
| 29 | No | 2 | 36 | 7 |
| 26 | No | 2 | 34 | 8 |
| 26 | Yes | 1 | 34 | 8 |
| 26 | No | 2 | 34 | 8 |
| 26 | Yes | 1 | 34 | 8 |
| 26 | No | 2 | 34 | 8 |
| 28 | Yes | 1 | 36 | 8 |
| 26 | No | 2 | 35 | 9 |
| 25 | No | 2 | 34 | 9 |
| 27 | No | 1 | 36 | 9 |
| 26 | No | 2 | 36 | 10 |
| 26 | No | 1 | 36 | 10 |
| 26 | No | 2 | 36 | 10 |
| 26 | No | 1 | 36 | 10 |
| 23 | No | 2 | 33 | 10 |
| 18 | No | 2 | 29 | 11 |
| 23 | No | 2 | 34 | 11 |
| 24 | No | 2 | 36 | 12 |
| 23 | No | 1 | 35 | 12 |
| 20 | Yes | 2 | 32 | 12 |
| 24 | No | 2 | 36 | 12 |
| 13 | No | 1 | 25 | 12 |
| 24 | Yes | 2 | 36 | 12 |
| 24 | No | 2 | 36 | 12 |
| 21 | No | 2 | 33 | 12 |
| 23 | No | 1 | 36 | 13 |
| 23 | Yes | 2 | 36 | 13 |
| 22 | No | 1 | 35 | 13 |
| 23 | No | 1 | 36 | 13 |
| 23 | No | 1 | 36 | 13 |
| 22 | Yes | 2 | 35 | 13 |
| 23 | No | 2 | 36 | 13 |
| 22 | Yes | 1 | 36 | 14 |
| 20 | Yes | 2 | 34 | 14 |
| 21 | Yes | 2 | 35 | 14 |
| 21 | No | 2 | 35 | 14 |
| 20 | No | 1 | 34 | 14 |
| 22 | Yes | 2 | 36 | 14 |
| 21 | No | 2 | 35 | 14 |
| 19 | No | 2 | 34 | 15 |
| 18 | No | 1 | 33 | 15 |
| 18 | Yes | 2 | 33 | 15 |
| 20 | No | 2 | 35 | 15 |
| 20 | No | 1 | 36 | 16 |
| 18 | No | 2 | 34 | 16 |
| 20 | No | 2 | 36 | 16 |
| **Last COVID-19 vaccination during pregnancy in** **gestational week** | **Vaccinated prior to pregnancy** | **Number of COVID-19 vaccines during pregnancy** | **Preterm labor in gestational week** | **Time between COVID-19 vaccine and PL (weeks)** |
| 15 | No | 1 | 31 | 16 |
| 20 | Yes | 2 | 36 | 16 |
| 19 | No | 1 | 35 | 16 |
| 18 | Yes | 2 | 34 | 16 |
| 20 | No | 2 | 36 | 16 |
| 18 | No | 2 | 35 | 17 |
| 19 | No | 2 | 36 | 17 |
| 17 | No | 2 | 34 | 17 |
| 18 | No | 2 | 35 | 17 |
| 11 | No | 1 | 28 | 17 |
| 16 | Yes | 1 | 33 | 17 |
| 19 | No | 2 | 36 | 17 |
| 17 | No | 2 | 34 | 17 |
| 17 | No | 2 | 35 | 18 |
| 18 | No | 2 | 36 | 18 |
| 18 | No | 2 | 36 | 18 |
| 11 | No | 1 | 29 | 18 |
| 16 | Yes | 2 | 34 | 18 |
| 18 | No | 2 | 36 | 18 |
| 13 | Yes | 2 | 32 | 19 |
| 17 | No | 2 | 36 | 19 |
| 11 | No | 2 | 30 | 19 |
| 16 | Yes | 2 | 36 | 20 |
| 15 | No | 1 | 35 | 20 |
| 15 | Yes | 1 | 35 | 20 |
| 14 | No | 1 | 34 | 20 |
| 13 | No | 1 | 34 | 21 |
| 15 | Yes | 1 | 36 | 21 |
| 14 | No | 1 | 35 | 21 |
| 11 | Yes | 2 | 32 | 21 |
| 11 | No | 1 | 32 | 21 |
| 13 | Yes | 1 | 35 | 22 |
| 14 | Yes | 2 | 36 | 22 |
| 11 | No | 1 | 33 | 22 |
| 13 | Yes | 2 | 36 | 23 |
| 12 | No | 1 | 35 | 23 |
| 10 | No | 1 | 33 | 23 |
| 9 | Yes | 2 | 32 | 23 |
| 10 | No | 2 | 35 | 25 |
| 5 | No | 1 | 31 | 26 |
| 9 | Yes | 2 | 35 | 26 |
| 7 | No | 2 | 33 | 26 |
| 6 | No | 1 | 34 | 28 |
| 4 | Yes | 1 | 34 | 30 |
| 3 | Yes | 2 | 35 | 32 |
| 4 | Yes | 2 | 36 | 32 |

**Table S3**. Overview of previous COVID-19 vaccinations, per trimester of pregnancy.

|  | Vaccinated in trimester 1  (N = 1,431) | Not vaccinated in trimester 1  (N = 4,453) | Vaccinated in trimester 2  (N = 3,059) | Not vaccinated in trimester 2  (N = 2,825) | Vaccinated in trimester 3  (N = 2,289) | Not vaccinated in trimester 3  (N = 3,595) |
| --- | --- | --- | --- | --- | --- | --- |
| No (previous) COVID-19 vaccination | 747 (52%) | 3,361 (75%) | 1,917 (63%) | 1,444 (51%) | 1,042 (46%) | 402 (11%) |
| Vaccinated prior to pregnancy ^a^ | 684 (48%) | 1,092 (25%) | 798 (26%) | 978 (35%) | 295 (13%) | 1,481 (41%) |
| Vaccinated in trimester 1^a^ | - | - | 408 (13%) | 1,023 (36%) | 467 (20%) | 964 (27%) |
| Vaccinated in trimester 2 ^a^ | - | - | - | - | 774 (34%) | 2,285 (64%) |

^a^ Vaccination prior to pregnancy, vaccination in trimester 1, and vaccination in trimester 2 are not mutually exclusive. A person can contribute to any of these groups.
